# Short Chain Fatty Acids Protect the Cognitive Function of Sepsis Associated Encephalopathy Mice via GPR43

**DOI:** 10.3389/fneur.2022.909436

**Published:** 2022-06-10

**Authors:** Hongsen Liao, Haojia Li, Hongguang Bao, Li Jiang, Jiayue Du, Yaoyi Guo, Yanna Si

**Affiliations:** ^1^Department of Anesthesiology, Nanjing First Hospital, Nanjing Medical University, Nanjing, China; ^2^Department of Ophthalmology, The Royal Wolverhampton NHS Trust, Wolverhampton, United Kingdom

**Keywords:** sepsis-associated encephalopathy (SAE), cecal ligation and puncture (CLP), short chain fatty acids (SCFAs), gut microbiota (GM), cognitive dysfunction

## Abstract

**Objective:**

This study aims to analyze the changes of fecal short chain fatty acids (SCFAs) content and gut microbiota composition in sepsis associated encephalopathy (SAE) mice, further evaluating the effect of SCFAs on cognitive function and the underlying mechanism in SAE mice.

**Methods:**

A total of 55 male adult C57BL/6 mice (2–3 months of age, 20–25 g) were divided into four groups randomly: sham group (*n* = 10), cecal ligation and puncture group (CLP group, *n* = 15), CLP+SCFAs group (*n* = 15), and CLP+SCFAs+GLPG0974 group (*n* = 15). Seven days after surgery, fecal samples were collected for microbiota composition and SCFA analysis from 6 mice in each group randomly. Behavioral test was applied to assess cognitive impairment at the same time. After that, mice were sacrificed and brain tissue was harvested for inflammatory cytokines analysis.

**Results:**

The levels of acetic acid (.57 ± 0.09 vs 2.00 ± 0.24, *p* < 0.001) and propionic acid (.32 ± 0.06 vs .66 ± 0.12, *p* = 0.002) were significantly decreased in the CLP group compared with the sham group. The administration of SCFAs significantly increased the levels of acetic acid (1.51 ± 0.12 vs. 0.57 ± 0.09, *p* < 0.001) and propionic acid (0.54 ± 0.03 vs. 0.32 ± 0.06, *p* = 0.033) in CLP+SCFAs group compared with CLP group. Relative abundance of SCFAs-producing bacteria, including *Allobaculum* (0.16 ± 0.14 vs. 15.21 ± 8.12, *p* = 0.037), *Bacteroides* (1.82 ± 0.38 vs. 15.21 ± 5.95, *p* = 0.002) and *Bifidobacterium* (0.16 ± 0.06 vs. 2.24 ± 0.48, *p* = 0.002), significantly decreased in the CLP group compared with the sham group. The behavioral tests suggested that cognitive function was impaired in SAE mice, which could be alleviated by SCFAs pretreatment. ELISA tests indicated that the levels of IL-1β, IL-6, and TNF-α were elevated in SAE mice and SCFAs could lower them. However, the GPR43 antagonist, GLPG0974, could reverse the cognitive protective effect and anti-neuroinflammation effect of SCFAs.

**Conclusion:**

Our study suggested that in SAE, the levels of acetate and propionate decreased significantly, accompanied by gut microbiota dysbiosis, particularly a decrease in SCFAs-producing bacteria. GPR43 was essential for the anti-neuroinflammation and cognitive protective effect of SCFAs in SAE.

## Introduction

Sepsis is a life-threatening syndrome with multiple organ dysfunction induced by a dysregulated host response to infection. It is still a severe global health problem with approximately 48.9 million sepsis diagnoses and 11.0 million sepsis-related deaths reported worldwide in 2017 (1). Sepsis associated encephalopathy (SAE) is a common complication of sepsis characterized by delirium with changes of the patient's consciousness in the acute phase and more than half of patients surviving sepsis develop long-term cognitive dysfunction (e.g., decreased learning ability, decreased memory) which impairs their quality of life severely (2, 3). Studies have shown that the levels of pro-inflammatory cytokines, such as interleukin (IL)-1β, IL-6, and tumor necrosis factor α (TNF-α), increase significantly in the hippocampus of a SAE mouse model, accompanied by microglia activation, which suggests that neuroinflammation is a possible pathological mechanism for SAE (3–5). Currently there are few effective clinical strategies to prevent or treat SAE.

Over the past decade, the gut microbiota has been considered to be closely related to the central nervous system and could influence neuro-inflammation through microbiota–gut–brain axis (6–8). This effect might play a role in SAE. Research have shown that SAE mice have severe gut microbiota dysbiosis, and regulation by means of probiotics could ameliorate the cognitive impairment induced by sepsis (9). Short-chain fatty acids (SCFAs), derived from intestinal microbial fermentation of indigestible dietary fiber, are important mediators of microbiota–gut–brain axis (10). SCFAs take part in many physiological processes including adipocyte differentiation, osteoblastic bone formation, and regulation of the inflammation (11). The anti-inflammatory effect of SCFAs has been proved to alleviate osteoarthritis, coronary heart disease, and perioperative neurocognitive disorders (12–14). Though recent study has shown that exogenous SCFAs administration ameliorated neuronal degeneration and blood–brain barrier disruption in SAE mice, the changes of SCFAs content in SAE is still unclear. Also the influence of SCFAs on cognitive function and the possible mechanism in SAE has not yet been elucidated (15).

G-protein-coupled receptor 43 (GPR43), or free fatty acid receptor 2 (FFAR2), exerts anti-inflammatory effect in the central nervous system when interacting with SCFAs. Acetate, one of the SCFAs, could suppress neuroinflammation and attenuate perioperative neurocognitive disorders by binding to GPR43 according to a recent research (14). The activation of GPR43 also ameliorates the accumulation of amyloid-β and neuroinflammation in Alzheimer disease (16). However, whether GPR43 exerts an anti-neuroinflammation effect by interacting with SCFAs in SAE or not is still unclear.

In this study, SCFAs (including acetate, propionate, and butyrate) and GPR43 antagonist GLPG0974 were administered on SAE mice models, which were established by cecal ligation and puncture (CLP) surgery. This study investigated the gut microbiota compositions and SCFAs detected. The levels of inflammatory cytokines of hippocampus were analyzed. Behavioral test was applied to assess cognitive impairment. Our present study and findings may provide new insights on the role of SCFAs in SAE with a postulated underlying mechanism.

## Materials and Methods

### Animals

A total of 55 male adult C57BL/6 mice (2–3 months of age, 20–25 g) were purchased from the animal core facility of Nanjing Medical University and kept in the barrier system. The mice were maintained under standard conditions: 12 h light/dark cycle, 22 ± 1 °C temperature, 52 ± 2% humidity, free access to food and water. The animal use protocol had been reviewed and approved by the Institutional Animal Care and Use Committee of Nanjing Medical University (IACUC-2109048).

### Cecal Ligation and Puncture (CLP) Procedure

Cecal ligation and puncture in rodents is considered as the gold standard in sepsis research (17). The model was established as previously described (18). Mice were anesthetized with sodium pentobarbital (40 mg/kg body weight, i.p.). After shaving and disinfection, a midline incision (1.5–2 cm) was made 1.5 cm below the xiphoid to gain access to the peritoneal cavity. The cecum was ligated at midpoint using 3–0 silk thread, then punctured two times at the midway between the ligation and cecal tip, and squeezed gently to empty the fecal contents into the peritoneal cavity. The ligated and punctured cecum was replaced back into the abdominal cavity before closing the peritoneum, abdominal muscle layer, and skin wound. In sham surgery, exteriorized cecum was replaced back into the abdominal cavity without ligation and puncture. Post-operatively, all mice were injected prewarmed (37 °C) sterile .9% saline (1 ml/mouse) subcutaneously to prevent hypovolemia.

### Drug Pretreatment and Experimental Design

**Figure 1:**
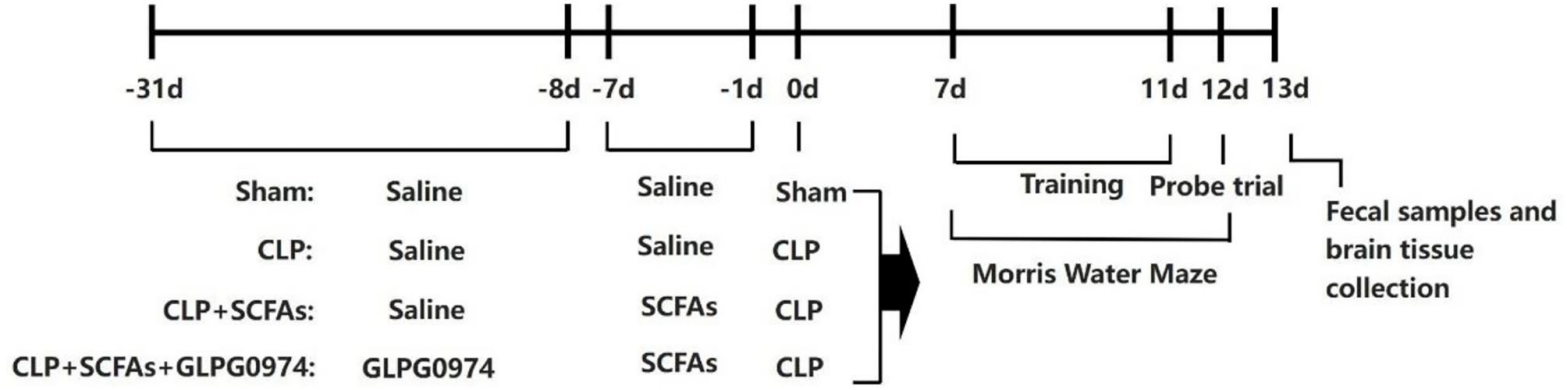
Schematic illustration of the experimental design. Short chain fatty acids (SCFAs) at 500 mg/kg body weight were administrated intra-gastrically two times a day for 7 consecutive days before the sham or cecal ligation and puncture (CLP) surgery, and GLPG0974 at 1 mg/kg body weight were given every 3 days for 24 days by oral gavage right before the administration of SCFAs. Cognitive function was assessed 7 days after surgery using a 6-day Morris water maze (MWM), after which the fecal samples and brain tissue were collected.

The mice were divided into four groups randomly: sham group (*n* = 10), CLP group (*n* = 15), CLP+SCFAs group (*n* = 15), and CLP+SCFAs+GLPG0974 group (*n* = 15). More mice were assigned to the last three groups to account for the expected mortality of CLP surgery (19). Several mice died during the experiment and the final sample size of each group was as follows: sham group (*n* = 10), CLP group (*n* = 10), CLP+SCFAs group (*n* = 12), and CLP+SCFAs+GLPG0974 group (*n* = 10). In the CLP+SCFAs+GLPG0974 group, SCFAs (acetate: propionate: butyrate at a ratio of 3: 1: 1) at 500 mg/kg body weight were administrated intra-gastrically two times a day for 7 consecutive days before CLP surgery, and GLPG0974 at 1 mg/kg body weight were given every 3 days for 24 days by oral gavage right before administration of SCFAs (15, 20). Saline was administrated as vehicle. In the CLP+SCFAs group, CLP surgery and SCFAs were given as described above, and an equal volume of saline was administered instead of GLPG0974. In the sham group and CLP group, only saline pretreatment was given before sham or CLP surgery. Seven days after surgery, fecal samples were collected for microbiota composition and SCFA analysis from 6 mice in each group randomly. Behavioral test was applied to assess cognitive impairment at the same time. After that, the mice were sacrificed and their brains were harvested for further experiment (Figure 1). SCFAs (acetate, propionate, and butyrate) were purchased from Aladdin, China. GLPG0974 were purchased from Tocris, United Kingdom.

### Morris Water Maze

Spatial learning and memory were assessed by the Morris water maze (MWM) test. A pool of opaque water (100 cm in diameter and 25 cm in depth, 23°C) with a hidden platform (10 cm in diameter) 1 cm below water surface at the center of one quadrant, and a video tracking and analyzing software (ANY-maze, Stoelting, USA) was used.

Between day 7 to day 11 post-operatively, mice were put through MWM 4 times on each training day, starting from a different quadrant each time, with an interval of 20 min between trials. Mice were consistently introduced facing the pool wall. A maximal latency of 60 s was given to reach the hidden platform. Those that failed to reach the platform within 60 s were manually guided to it. After staying on the platform for 10 s, the mice were dried and transferred to the cage. The latency to the platform and path taken were recorded for each mouse. If the mouse failed to reach the platform within 60 s, the latency was recorded as 60 s.

A probe trial was performed on day 12 post-operatively without the submerged platform. The time spent in the target quadrant and platform crossovers were recorded.

### Gut Microbiota Composition Analysis

A total of 1,000 mg fecal pellets were collected in sterile tubes for each mouse. Microbiota DNA was extracted using the QIAamp DNA Stool Mini Kit (Qiagen, USA) and applied to amplification of V3–V4 regions of 16S rDNA. PCR amplification of the bacterial 16S rRNA genes V3-V4 region was performed using the forward primer 338F (5'-ACTCCTACGGGAGGCAGCA-3') and the reverse primer 806R (5'-GGACTACHVGGGTWTCTAAT-3'). PCR amplicons were purified with Vazyme VAHTSTM DNA Clean Beads (Vazyme, Nanjing, China) and quantified using the Quant-iT PicoGreen dsDNA Assay Kit (Invitrogen, Carlsbad, CA, USA). After individual quantification, amplicons were pooled in equal amounts, and pair-end 2 × 250 bp sequencing was performed using the Illlumina MiSeq platform with MiSeq Reagent Kit v3 at Shanghai Personal Biotechnology Co., Ltd., (Shanghai, China).

### Sequence Analysis

Microbiome bioinformatics were performed with QIIME2 2019.4. Briefly, raw sequence data were demultiplexed using the demux plugin followed by primers cutting with cutadapt plugin (21). Sequences were then quality filtered, denoised, merged, and chimera removed using the DADA2 plugin (22). Non-singleton amplicon sequence variants (ASVs) were aligned with mafft and used to construct a phylogeny with fasttree2 (23, 24). A total number of 867,552 clean reads were obtained. Taxonomy was assigned to ASVs using the classify-sklearn naïve Bayes taxonomy classifier in feature-classifier plugin against the SILVA Release 132 Database (25).

### SCFAs Analysis

Gas Chromatography-Mass Spectrometer (GC-MS) analysis was used to quantify SCFAs in fecal samples. The sample preparation and GC-MS analysis were performed as described previously (26, 27). Twenty milligrams of fecal samples were accurately weighed and placed in a 2 ml EP tube. One milliliter of phosphoric acid (.5% v/v) solution and a small steel ball were added to the EP tube. The mixture was grinded for 10 s three times, then vortexed for 10 min and ultrasonicated for 5 min. The mixture was centrifuged at 12,000 r/min at 4°C, with 0.1 ml of supernatant added at 10 min. Then, 0.5 mL MTBE (containing internal standard) solution was added. The mixture was vortexed for 3 min and ultrasonicated for 5 min. The mixture was further centrifuged at 12,000 r/min for 10 min at 4°C. The supernatant was collected and used for GC-MS analysis. Agilent 7890 B gas chromatograph coupled to a 7000 D mass spectrometer with a DB-FFAP column (30 m length × .25 mm i.d. × .25 μm film thickness, J&W Scientific, USA) was employed for GC-MS analysis of SCFAs. Helium (flow rate 1.2 ml/min) was used as carrier gas. A 2 μL injection was made in the split mode. The oven temperature was held at 90°C for 1 min, raised to 100°C at a rate of 25°C/min, then raised to 150°C at a rate of 20°C/min, held for .6 min, raised to 200°C at a rate of 25°C/min, then held for .5 min after running for 3 min. All samples were analyzed in multiple reaction monitoring mode. The injector inlet and transfer line temperature were 200°C and 230°C, respectively.

### Enzyme-Linked Immunosorbent Assay (ELISA)

The hippocampus samples from 6 mice in each group were collected after the behavioral tests, and the level of IL-1β, IL-6, and TNF-α in hippocampus was detected by mouse IL-1β, IL-6, or TNF-α ELISA kits according to the instructions. A monoclonal antibody specific for mouse IL-1β, IL-6, or TNF-α was briefly coated onto the microplates. Wells were incubated for 2 h at room temperature with test samples (hippocampus tissue) and washed five times. Then, 100 μL of mouse IL-1β, IL-6, or TNF-α conjugate was added to each well and incubated further for 2 h. The washing was repeated two times. Wells were then incubated in 100 μL of substrate solution for 30 min and stopped with stop solution (100 μL). Determination of the optical density of each well was set at 450 nm and corrected at 570 nm. A standard curve was constructed using various dilutions of TNF-α, IL-6, and IL-1β standard preparation. The levels of cytokines were calculated according to standard curves. Three replicates were used for ELISA plates to reduce errors. Mouse IL-1β, IL-6, and TNF-α ELISA kits were purchased from Abcam, United States.

### Statistical Analysis

All data were expressed as the mean ± standard error of the mean (SEM). Between-group comparisons were analyzed using one-way ANOVA followed by LSD *post hoc* test for multiple comparisons on normally distributed data. Otherwise, non-parametric Kruskal-Wallis was used. Data from Morris water maze training were analyzed using two-way ANOVA, followed by LSD post hoc test for multiple comparisons (SPSS 20.0 software). The Kaplan-Meier method was used to estimate the survival rate, which was compared by the log-rank test. A value of *p* < 0.05 was considered significant.

Sequence data analyses were mainly performed using QIIME2 and R packages (v3.2.0). After rarefaction at 6,000 reads per sample, alpha-diversity metrics were calculated in QIIME2. The linear discriminant-analysis effect size (LEfSe) was further used to identify the dominant bacteria taxa in four groups with the default parameters using R packages (v3.2.0) (28).

## Results

### The Levels of Acetate and Propionate Decreased in SAE Mice

**Figure 2:**
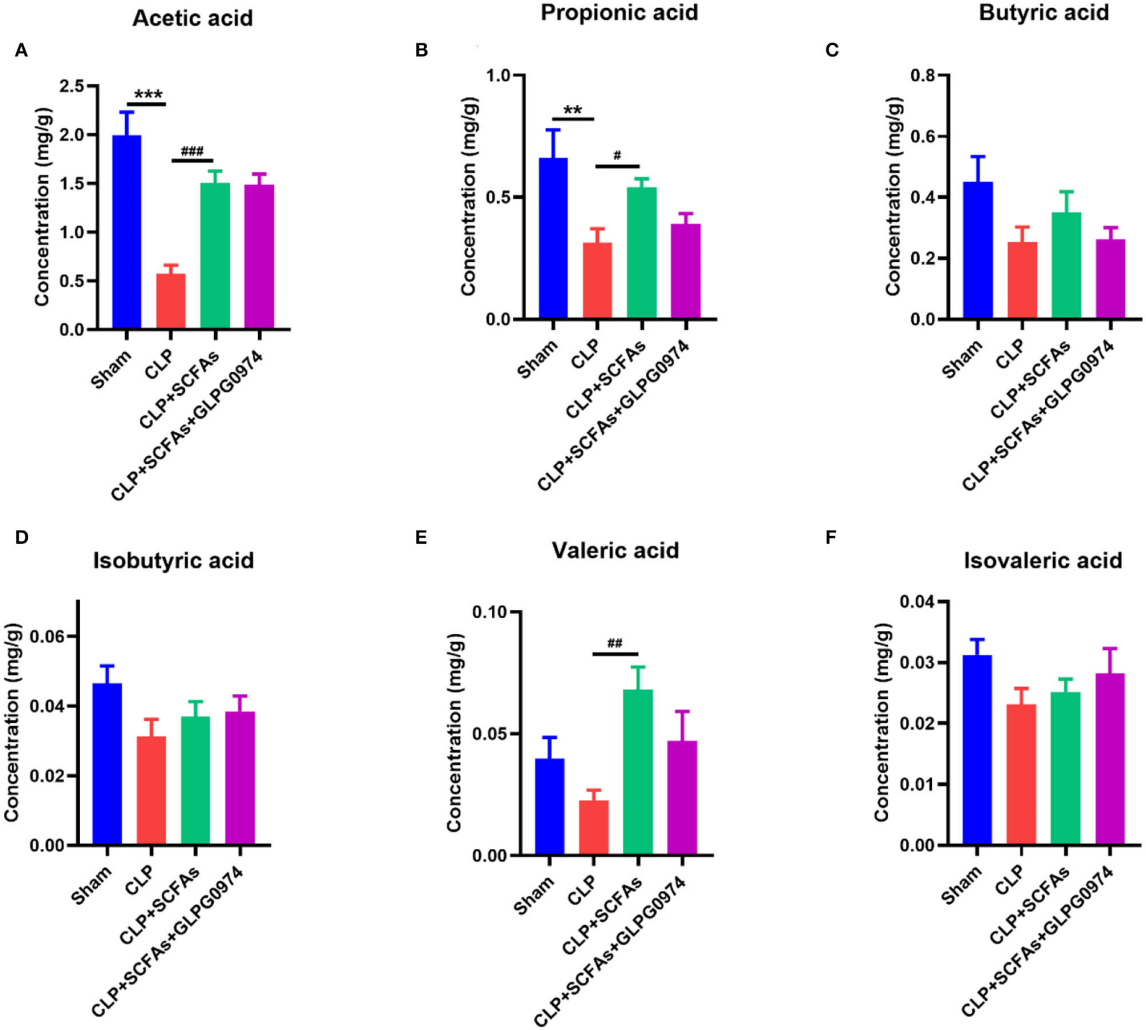
Fecal SCFAs content of four groups (*n* = 6 per group). The levels of **(A)** acetic acid, **(B)** propionic acid **(C)** butyric acid **(D)** isobutyric acid **(E)** valeric acid, and **(F)** isovaleric acid were analyzed. **p < 0.01, ***p < 0.001 vs. sham group, #*p* < 0.05, ##*p* < 0.01, ###*p* <0.001 vs. CLP group.

Short chain fatty acids, as a class of key bacterial metabolites, show anti-inflammatory effect in many diseases. In this study, six major SCFAs (acetic acid, propionic acid, butyric acid, isobutyric acid, valeric acid, and isovaleric acid) were measured in fecal samples of the four groups (*n* = 6). The levels of acetic acid (Figure 2A, *p* < 0.001) and propionic acid (Figure 2B, *p* = 0.003) were significantly decreased in the CLP group compared with the sham group, while the levels of butyric acid, isobutyric acid, valeric acid, and isovaleric acid were not influenced (Figures 2C-F). SCFAs pre-treatment significantly increased the levels of acetic acid (Figure 2A, *p* < 0.001), propionic acid (Figure 2B, *p* = 0.020), and valeric acid (Figure 2E, *p* = 0.002) in CLP+SCFAs group compared with CLP group, while no statistical differences were found in other kinds of SCFAs (Figures 2C,D,F). No significant difference was shown between CLP+SCFAs group and CLP+SCFAs+GLPG0974 group (Figure 2).

### SCFAs-Producing Bacteria Decreased in SAE Mice

**Figure 3:**
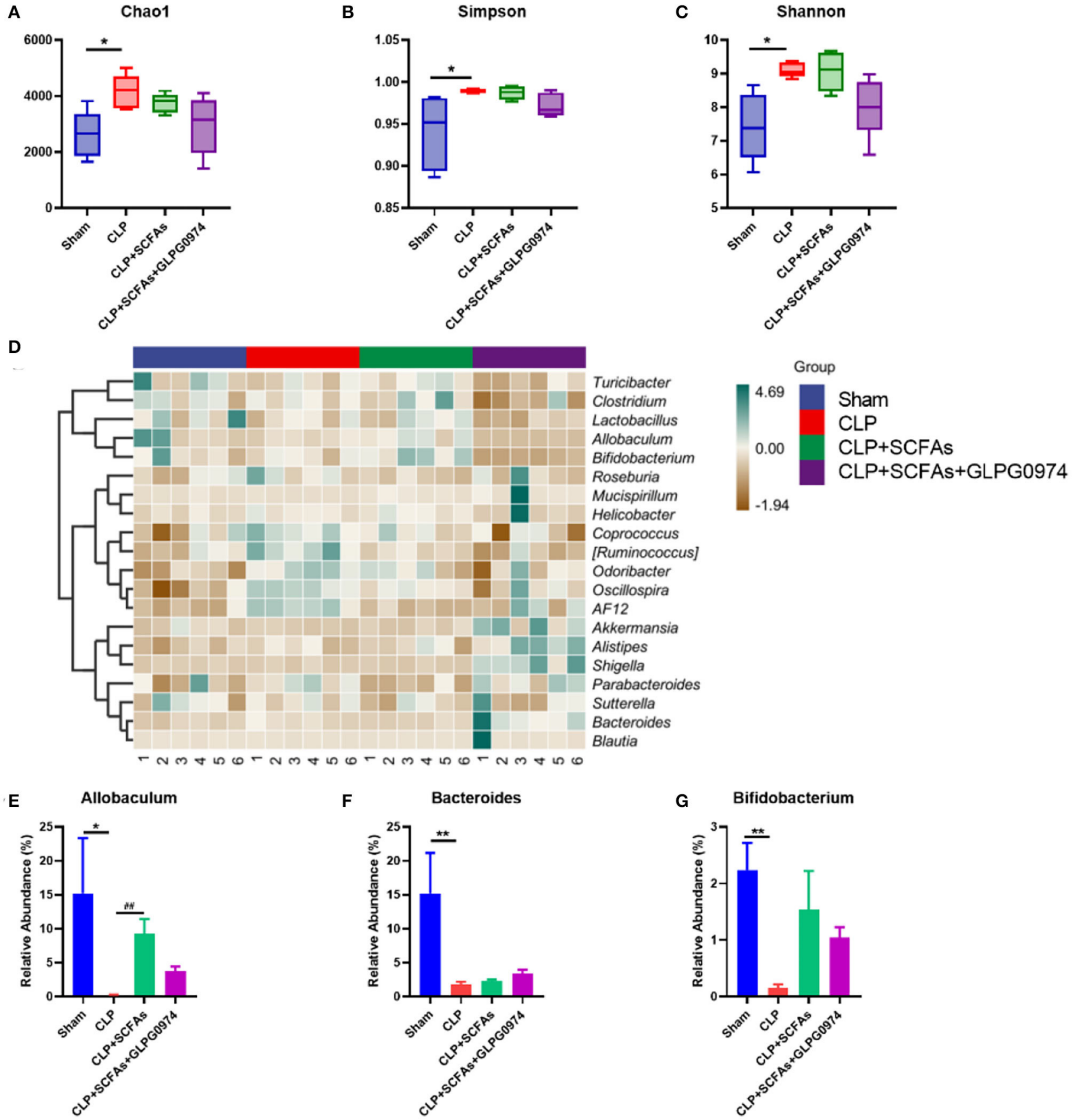
Changes of gut microbiota composition among four groups (*n* = 6 per group). **(A–C)** α diversity index of the four groups **(A)** Chao1 index **(B)** Simpson index **(C)** Shannon index **(D)** Gut microbiota composition heatmap of the four groups on genus level **(E–G)** Relative abundance of SCFAs-producing bacteria in four groups **(E)** *Allobaculum* **(F)** *Bacteroides* **(G)** *Bifidobacterium*. *p < 0.05, **p < 0.01 vs. sham group, ##*p* <0.01 vs. CLP group.

Gut microbiota was analyzed among the four groups (*n* = 6). The results of alpha diversity demonstrated that chao1 (Figure 3A, *p* = 0.026), Simpson (Figure 3B, *p* = 0.02) and Shannon (Figure 3C, *p* = 0.017) index were significantly different in the CLP group compared with the sham group.

In order to further study differences in gut microbiota composition, the top 20 bacteria taxa on genus level with the highest relative abundance were analyzed (Figure 3D). Among them, SCFAs-producing bacteria such as *Allobaculum* (Figure 3E, *p* = 0.037), *Bacteroides* (Figure 3F, *p* = 0.002) and *Bifidobacterium* (Figure 3G, *p* = 0.002) was significantly reduced in the CLP group compared with the sham group. *Allobaculum* (Figure 3E, *p* = 0.002) was significantly increased in the CLP+SCFAs group compared to the CLP group, while no significant differences were found in *Bacteroides* (Figure 3F) and *Bifidobacterium* (Figure 3G). The levels of SCFAs-producing bacteria were not influenced by GLPG0974 as no significant differences were found between CLP+SCFAs group and CLP+SCFAs+GLPG0974 group (Figures 3E-G).

**Figure 4:**
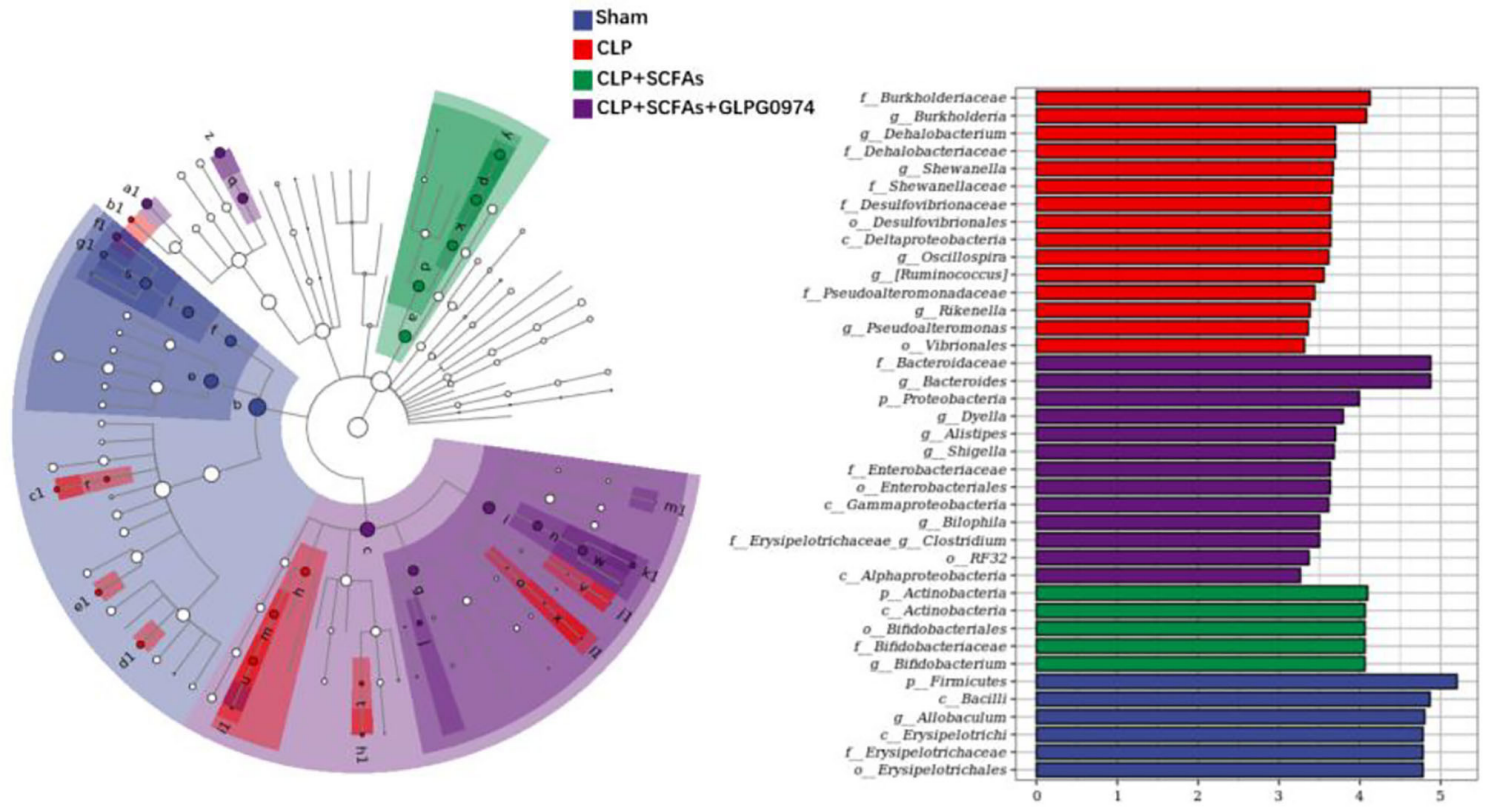
Dominant bacteria taxa in four groups were analyzed using linear discriminant-analysis effect size (LEfSe) (*n* = 6 per group). A total of 39 gut microbiota taxa with the greatest differences in the abundance among four groups were identified.

Furthermore, LEfSe analysis was used to identify the dominant bacteria taxa in different groups. A total of 39 bacteria taxa with statistically significant and biologically consistent differences were found (Figure 4). The phylum *Firmicutes*, class *Bacilli*, genus *Allobaculum*, etc. were significantly enriched in the sham group. While the family *Burkholderiaceae*, genus *Burkholderia*, genus *Dehalobacterium*, etc. were most likely to explain the differences between the CLP group and the other mice. In the CLP+SCFAs group, phylum *Actinobacteria*, class *Actinobacteria*, order *Bifidobacteriales*, etc. were significantly increased. In the CLP+SCFAs+GLPG0974 group, the family *Bacteroidaceae*, genus *Bacteroides*, phylum *Proteobacteria*, etc. were uniquely enriched. Among them, *Allobaculum, Bifidobacterium*, and *Bacteroides* were more abundant in the sham, CLP+SCFAs or CLP+SCFAs+GLPG0974 groups.

### SCFAs Inhibited Sepsis-Induced Cognitive Dysfunction via GPR43

Morris water maze was performed to analyze the cognitive functionof each group at 7 days post-operatively.

**Figure 5:**
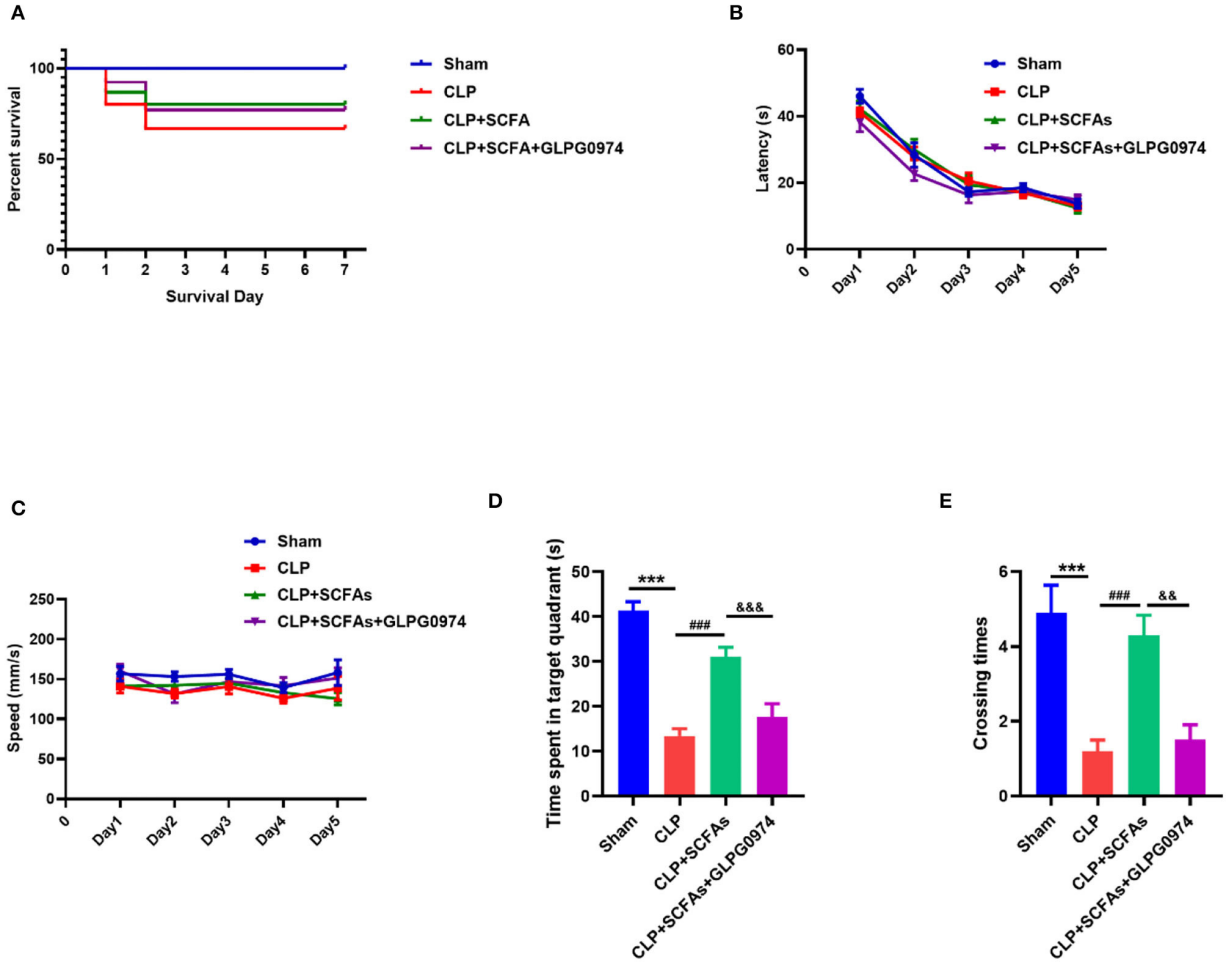
Survival rate and behavioral tests results. **(A)** Survival curves of four groups **(B–E)** MWM test results (*n* = 10 per group) **(B)** The latency of finding the hidden platform in training days **(C)** the average swimming speed in training days **(D)** the time spent in the target quadrant in probe trail, and **(E)** The number of crossings of the platform location. ***p < 0.001 vs. sham group, ###*p* < 0.001 vs. CLP group, &&*p* < 0.01, &&&*p* < 0.001 vs. CLP+SCFAs group.

Two mice died due to gavage in the CLP+SCFAs+GLPG0974 group. The post-operative 7-day survival rates of mice in the sham, CLP, CLP+SCFAs, and CLP+SCFAs+GLPG0974 groups were 100.0% (10/10), 66.7% (10/15), 80.0% (12/15), and 76.9% (10/13), respectively (Figure 5A). Two mice in the CLP+SCFAs group were excluded from MWM due to poor wound healing. No mortality was reported during behavioral testing.

In the MWM, there was no significant between-group difference in latency and average speed in the training phase (Figures 5B,C), which indicates that all mice had learnt to achieve the exercise goal after 5 days of training, and mobility was not affected by sepsis. However, in the probe trial, the CLP group spent less time in the target quadrant (Figure 5D, *p* < 0.001) and had fewer crossings in the target quadrant (Figure 5E, *p* < 0.001) than the sham group. In contrast, the CLP+SCFAs group spent more time in the target quadrant (Figure 5D, *p* < 0.001) and had more crossings (Figure 5E, *p* < 0.001) in the target quadrant compared with the CLP group, and GLPG0974 reversed these changes (Figure 5D, *p* < 0.001, Figure 5E, *p* = 0.001).

### SCFAs Attenuated Neuroinflammation in SAE Mice *via* GPR43

**Figure 6:**
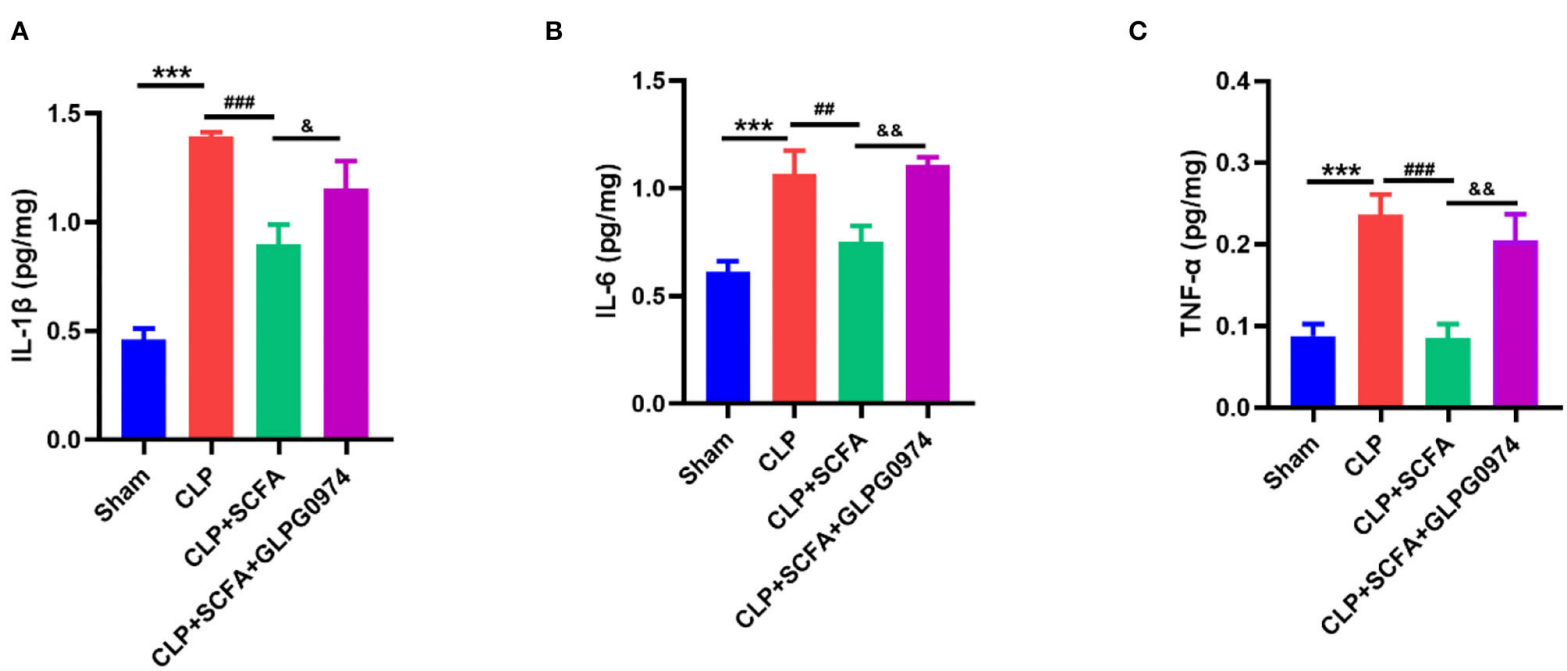
The levels of IL-6, IL-1β, and TNF- α in the hippocampus of the mice (*n* = 6 per group). **(A)** IL-1β, **(B)** IL-6, **(C)** TNF-*upalpha*. ***p < 0.001 vs. sham group, ##*p* < 0.01, ###*p* < 0.001 vs. CLP group, &*p* < 0.05, &&*p* < 0.01 vs. CLP+SCFAs group.

The levels of several inflammatory cytokines in the hippocampus were measured to evaluate neuro-inflammation. Compared with the sham group, the levels of IL-1β (Figure 6A, *p* < 0.001), IL-6 (Figure 6B, *p* < 0.001) and TNF-α (Figure 6C, *p* < 0.001) were significantly increased in CLP group. Conversely, the levels of IL-1β (Figure 6A, *p* < 0.001), IL-6 (Figure 6B, *p* = 0.006) and TNF-α (Figure 6C, *p* < 0.001) were significantly lower in the CLP+SCFAs group than those in the CLP group. GLPG0974 reversed the anti-neuroinflammatory effect of SCFAs, by increasing IL-1β (Figure 6A, *p* = 0.038), IL-6 (Figure 6B, *p* = 0.002), and TNF-α (Figure 6C, *p* = 0.002) significantly in the CLP+SCFAs+ GLPG0974 group compared with the CLP+SCFAs group.

## Discussion

In this study, we measured the SCFAs content and analyzed the gut microbiota composition in SAE mice and explored the mechanism of anti-neuro-inflammatory and cognitive protective effect of SCFAs by administrating SCFAs and GPR43 antagonist in SAE mice. We found that SAE mice had lower levels of SCFAs, especially acetate and propionate. Gut microbiota dysbiosis existed in SAE mice, and SCFAs-producing bacteria, including *Allobaculum, Bacteroides*, and *Bifidobacterium*, were significantly decreased in SAE mice. Conversely, the administration of SCFAs could alleviate neuro-inflammation and cognitive dysfunction in SAE, in which effects could be reversed by GPR43 antagonist.

Sepsis associated encephalopathy is a critical disease with high incidence and high mortality (3). Cognitive dysfunction in SAE is both an important acute symptom and a well-recognized long-term complication. Cognitive impairment gives rise to altered memory, attention, and concentration, which severely affects employment and everyday life (29). In this study, medium cecal ligation and puncture surgery, an established and comparable method, was used to establish sepsis in mice model (19). MWM test was used to evaluate hippocampus-dependent learning and memory, which are important aspects of cognitive function associated with sepsis (30). Swimming speed was recorded to exclude the influence of reduced spontaneous locomotor movement in the MWM (31). SAE mice spent less time in the target quadrant and had fewer crossings in the target quadrant, which was consistent with our previous study (5). These results suggest that CLP may cause cognitive dysfunction and mimic key features of SAE.

Cognitive impairment in SAE is associated with neuro-inflammation (2). As described by previous research, SAE mice underwent significant neuro-inflammation due to increased levels of pro-inflammatory cytokines such as IL-1β, IL-6, whereas down-regulation of CXCR5 could alleviate cognitive dysfunction by inhibiting neuro-inflammation in SAE (32). SCFAs administration has been described to inhibit neuro-inflammation in many central nervous system diseases (14, 33, 34). Recent research demonstrates that the administration of SCFAs ameliorated neuronal degeneration and blood–brain-barrier disruption in SAE mice (15). With a focus on changes in cognitive function after SCFAs administration, we observed that the neuro-protective effect of SCFAs also contributed to cognitive recovery in SAE mice.

Although SCFAs demonstrated protective effects in SAE, to our knowledge, fecal SCFAs content has not been reported yet in SAE. Herein we confirmed that SCFAs, especially acetic acid and propionic acid, was significantly decreased in SAE mice, and the administration of SCFAs may increase SCFAs content effectively. As SCFAs are metabolites of gut microbiota, we further analyzed the gut microbiota composition. The gut microbiota dysbiosis found in SAE mice was partly consistent with previous research, for example, more abundant *Pseudomonas* in SAE mice (35). Besides, *Burkholderia* also increased in the CLP group, which was reported to be associated with neonatal sepsis in a recent research (36). In contrast, *Clostridium* enriched in the CLP+SCFAs+GLPG0974 group exerts neuroprotective effect in traumatic brain injury *via* the gut-brain axis, according to another research (37). As expected, SCFAs-producing bacteria including *Allobaculum, Bifidobacterium*, and *Bacteroides* was significantly decreased in SAE mice compared with the sham group. The administration of SCFAs improved both SCFAs content and gut microbiota dysbiosis, thereby increasing the levels of SCFAs-producing bacteria. This suggests that there may be a complex interaction between gut microbiota and SCFAs content.

The possible mechanism of cognitive function protection of SCFAs was then explored. Free fatty acid receptors (FFARs) are a series of G protein-coupled receptors (GPCRs) that exert key physiological functions by binding with free fatty acids (11). GPR43 is one of the FFARs activated by SCFAs, e.g., acetate, propionate, and butyrate (38). GPR43 is important for macrophages to transform into anti-inflammatory M2-type (39). Research has found that acetate could attenuate neuro-inflammation in the hippocampus of aged mice and therefore alleviate peri-operative neuro-cognitive disorders, whereas silencing GPR43 in BV2 microglia partially reversed the anti-neuro-inflammatory effect of acetate (14). In this study, GPR43 antagonist was administered to verify the effect of GPR43 in SAE *in vivo*. Results suggest that the blockade of GPR43 canceled the protective effect of SCFAs on both cognitive function and neuro-inflammation. Furthermore, GLPG0974 did not negatively affect SCFAs content or SCFAs-producing bacteria, as no difference was observed between CLP+SCFAs group and CLP+SCFAs+GLPG0974 group.

Recent research indicates that GPR43/β-arrestin-2/NF-κB downstream signaling exerts anti-inflammatory effects in diabetic nephropathy and lipopolysaccharide-induced liver injury (40, 41). β-arrestin-2 is a widely expressed protein which plays an important role in the desensitization and internalization of G-protein-coupled receptors (GPCRs) (42). SCFAs could activate GPR43, which in turns promotes interaction between β-arrestin-2 and I-κBα and therefore inhibits NF-κB signaling (40, 43). GPR43/β-arrestin-2/NF-κB is the potential mechanism for the protective effect of SCFAs in SAE, since we have previously demonstrated NF-κB activation in SAE mice (32). Results from this study provides potential research direction for more detailed experiments to fully illustrate the mechanism, such as co-immuno-precipitation to identify the exact interaction between β-arrestin-2 and I-κBα.

This study has some limitations. First, GPR43 antagonist was administered, whereas GPR43 deficient mice may provide stronger evidence for the mechanism. Secondly, SCFAs concentration in the brain was not measured. Thirdly, the mixture of SCFAs (including acetate, propionate, and butyrate) was used as pre-treatment in accordance with the existing study (15). With our data on decreased levels of acetate and propionate in SAE mice, acetate or propionate pre-treatment could be administered in a future study to verify the effect of individual SCFAs. Finally, the effect of SCFAs administration after CLP surgery needs to be evaluated in the next study.

## Conclusion

Our study describes decreased levels of acetate and propionate, and a reduction in SCFAs-producing bacteria in SAE. We demonstrate that GPR43 is essential to the anti-neuroinflammatory and cognitive-protective effects of SCFAs in SAE.

## Data Availability Statement

The datasets presented in this study can be found in online repositories. The names of the repository/repositories and accession number(s) can be found below: National Center for Biotechnology Information (NCBI) BioProject, https://www.ncbi.nlm.nih.gov/bioproject/, PRJNA823481 and NCBI BioSample, https://www.ncbi.nlm.nih.gov/biosample, SAMN27302389-SAMN27302412.

## Ethics Statement

The animal study was reviewed and approved by Institutional Animal Care and Use Committee of Nanjing medical university.

## Author Contributions

HLia, and HB designed the research. HLia, HLi, JD, and YG performed the research. HLia collected and analyzed the data. YS and HB provided administrative support. HLia, HB, and LJ drafted the manuscript. All authors listed have made a substantial, direct, and intellectual contribution to the work and approved it for publication. All authors contributed to the article and approved the submitted version.

## Funding

This work was supported by grants from the National Natural Science Foundation of China (grant numbers: 81971872 and 81873954).

## Conflict of Interest

The authors declare that the research was conducted in the absence of any commercial or financial relationships that could be construed as a potential conflict of interest.

## Publisher's Note

All claims expressed in this article are solely those of the authors and do not necessarily represent those of their affiliated organizations, or those of the publisher, the editors and the reviewers. Any product that may be evaluated in this article, or claim that may be made by its manufacturer, is not guaranteed or endorsed by the publisher.
